# Ursodeoxycholic acid versus phenobarbital for cholestasis in the Neonatal Intensive Care Unit

**DOI:** 10.1186/s12887-018-1167-y

**Published:** 2018-06-20

**Authors:** Tamorah Lewis, Simisola Kuye, Ashley Sherman

**Affiliations:** 1 Department of Pediatrics, Children’s Mercy Hospital, University of Missouri Kansas City School of Medicine, 2401 Gillham Rd, Kansas City, MO 64108 USA; 2 School of Medicine, University of Missouri Kansas City, Kansas City, MO USA

**Keywords:** Neonate, Cholestatic jaundice, Ursodiol, Phenobarbital, Neonatal intensive care unit

## Abstract

**Background:**

Although neonates and young infants with cholestasis are commonly treated with either phenobarbital or ursodeoxycholic acid (ursodiol), there is no evidence that phenobarbital is effective for this indication. Our objective was to compare the effectiveness of ursodiol and phenobarbital for the treatment of cholestasis in a diverse NICU population.

**Methods:**

This is a retrospective cohort study including infants with cholestasis who were admitted to a Level IV NICU between January 2010 and December 2015. Drug courses of phenobarbital and ursodiol were identified within the medical record, and medical, demographic, and drug information were extracted. The primary outcome was reduction in direct bilirubin.

**Results:**

Sixty-eight infants provided a total of 112 courses of drug therapy for comparison. Diverse medical diagnoses were captured in the patient cohort. Ursodiol was significantly more effective in reducing direct bilirubin than was phenobarbital (− 1.89 vs + 0.76 mg/dL; − 33.33 vs + 13.0 umol/L, *p*-value 0.03), even after controlling for baseline cholestasis severity, intrauterine growth restriction status, and lipid lowering therapy (− 2.16 vs + 0.27 mg/dl; − 36.94 vs + 4.62 umol/L, *p*-value 0.03). There was no improvement in direct bilirubin in the majority of infants treated with phenobarbital.

**Conclusions:**

Phenobarbital, as compared to ursodiol, has limited efficacy for the reduction of direct bilirubin in neonates and young infants with cholestasis. Given new data regarding the potential neurotoxicity of phenobarbital in the developing brain, providers may choose to avoid phenobarbital in the treatment of cholestasis in infants.

## Background

Hepatic cholestasis is the result of impaired balance between bile acid uptake and efflux. Abnormal hepatic accumulation of bile salts leads to disruption of cell membranes and cellular organelles resulting in necrosis, inflammation, and fibrosis. Cholestasis is commonly encountered in the neonatal intensive care unit (NICU) as a result of multiple medical conditions, including extreme prematurity, growth restriction, and sepsis [1]. Additionally, congenital anomalies of the gastrointestinal tract requiring surgery and other medical conditions requiring prolonged parenteral nutrition are associated with cholestasis. Given the known hepatotoxic effects of bile salt stasis, physicians often treat cholestasis with multiple modalities, including IV lipid limiting [2] and the medications ursodiol (ursodeoxycholic acid) or phenobarbital.

Ursodiol is the only drug approved by the Food and Drug Administration for use in adult cholestatic conditions. Ursodiol protects injured cholangiocytes against the toxic effects of bile acids and stimulates bile acid secretion via calcium-dependent mechanisms. Additionally, it directly modulates transcription of transporters and inhibits bile-acid induced hepatocyte apoptosis [3, 4]. Ursodiol is proven effective at treating parenteral nutrition-associated cholestasis in small cohorts of infants [5, 6], but is not used as prophylaxis in high-risk neonates [7, 8].

Phenobarbital acts via the nuclear receptor called constitutive androstane receptor (CAR), controlling hepatocellular metabolizing enzymes and transporters. Phenobarbital improves cholestasis in a small cohort of majority adult patients with anatomic abnormalities, including primary biliary cirrhosis, sclerosing cholangitis, and intrahepatic biliary hypoplasia [9], but there is no evidence that it is effective at treating neonatal cholestasis. There is recent concern that phenobarbital carries more risk than previously appreciated. Specifically, animal models of the developing brain have shown that phenobarbital leads to neuronal apoptosis [10, 11] and long-term behavioral toxicity [12].

Given the limited evidence for efficacy and increased evidence for toxicity with phenobarbital, we aimed to compare the two drugs ursodiol and phenobarbital for effectiveness in treatment of cholestasis in a diverse cohort of infants. We hypothesized that ursodiol would be more effective at reducing direct bilirubin (DBili), a surrogate marker for hepatic cholestasis. Our primary outcome was change in direct bilirubin, with a secondary outcome of direct bilirubin at the end of drug therapy.

## Methods

This retrospective cohort study was approved by the Children’s Mercy Institutional Review Board prior to data extraction. Eligible infants between January 2010 and December 2015 were identified using the variable “highest direct bilirubin” in the Children’s Hospitals Neonatal Database (CHND), and all children admitted to the Children’s Mercy Intensive Care Nursery with a documented direct bilirubin > 3.0 mg/dl (51.34 umol/) were identified to eliminate cases of mild cholestasis. Infants were randomly selected from this list, without knowledge of drug treatment or bilirubin changes, for inclusion in the study cohort. Infants included in the study were treated with ursodiol and/or phenobarbital per standard clinical care in the NICU, at the discretion of the neonatologist at the time of treatment. There is no protocol which dictates drug treatment for cholestasis, but most practitioners use phenobarbital while an infant is not enterally fed and ursodiol when on feeds. Because we could not extract important study data from outside charts, infants were excluded if they were transferred to our NICU already on medication for cholestasis, or if they were discharged to another NICU on cholestasis medications.

### Study variables

Because many infants were exposed to more than one course of medication treatment for cholestasis, we defined the research unit as a course of medication administration, e.g. ursodiol for 25 days. All relevant variables were then extracted for that medication course, including direct bilirubin levels when the medication was started and stopped. If the direct bilirubin was measured a few days before stopping the medication, we used the date of the last bilirubin measurement as the stop date. If drug treatments overlapped, we only used data until the day before the second drug was added in order to exclude overlapping drug effect. Medication courses were excluded if they were less than 1 week long because we felt that a meaningful change in cholestasis could not be appreciated with such a short treatment course.

Demographic data (gestational age, race, gender, birthweight), major medical diagnoses, and drug data (daily dose, length of therapy) were collected from the charts of eligible infants. Data about potential confounding variables including nutrition (lipid lowering treatment, age when enteral nutrition achieved) were extracted from each patient chart. Lipid lowering refers to the practice of decreasing parenteral intralipid administration from 3 mg/kg/day to 1 mg/kg/day and is used as first line treatment of TPN-associated cholestasis in the NICU where this study was performed. Some infants with cholestasis with not be treated with lipid lowering if they have relative contraindications such as poor growth or inability to tolerate higher glucose infusion rates or protein to supplement calories lost in lipid lowering.

### Statistical analysis

To calculate sample size, we assumed based on clinical experience that ursodiol would have a much larger effect on improving direct bilirubin than would phenobarbital (average improvement in DBili 3 mg/dL; 51.34 umol/L vs 0.3 mg/dL; 5.13 umol/L), with a standard deviation of 4 mg/dL (68.45 umol/L). Using a two-sided significance value of .05 and a two group t-test, an N of 35 in each group provides 80% power to detect a difference.

Two analyses were performed. The first analysis included only the first course of drug therapy to eliminate any carryover effect from treatment with a prior medication for cholestasis. The second analysis included all drug courses, and included a variable to account for prior drug therapy within a 14-day window. Categorical variables were compared between the two drug treatment groups using Chi-squared analysis. Continuous variables were compared between the two drug groups using Wilcoxon rank sums analysis. Univariate analysis of the primary outcome of change in direct bilirubin was performed using analysis of variance (ANOVA), and multivariate analysis was performed using mixed effect regression modeling. We controlled for confounders including direct bilirubin at start of therapy (baseline severity), intrauterine growth restriction (IUGR), and lipid lowering (a commonly used alternative treatment for cholestasis). Although these variables were not statistically significantly different between the drug groups, there is biological plausibility that these clinical confounders could affect the scientific comparison. All statistical analyses were performed using SAS version 9.4.

Two infants with extreme phenotypes were excluded: (1) a preterm, IUGR infant with congenital diaphragmatic hernia who died on extracorporeal membrane oxygenation (ECMO) at day 14 with a direct bilirubin of 48 who was treated with phenobarbital, and (2) a full-term infant with congenital leukemia who developed cholestasis as a chemotherapy side effect in whom anti-cholestatic medications were used prophylactically.

## Results

Data on 68 infants were extracted to obtain 68 first courses of drug therapy for comparison and 112 total courses of drug therapy for comparison. 49% of patients had one eligible drug course, 40% had two eligible courses, and 11% had more than two eligible courses. The primary medical diagnoses of the patients in the cohort are listed in Table 1.

**Table 1:** Patient cohort medical diagnoses

Diagnosis	N (68)	ECMO
Preterm, Uncomplicated	11	
Preterm, Sepsis	7	1
Preterm, Spontaneous Intestinal Perforation	2	
Surgical NEC	12	
Medical NEC	7	
Congenital Heart Disease^a^	6	
Congenital Diaphragmatic Hernia (CDH)	4	2
Malrotation ± Bowel Atresia	4	
Gastroschisis / Omphalocele	3	
Other	12	2

Other: 1 Biliary atresia, 3 fullterm with pneumonia/sepsis (2 on ECMO), 3 hepatitis / liver failure of unknown etiology, 1 congenital CMV, 1 homocystinuria, 1 undiagnosed genetic syndrome with multiple anomalies, 1 panhypopituitarism with multiple anomalies, 1 preterm with congenital lung malformation

^a^one with documented medical NEC. NEC = necrotizing enterocolitis

The gestational ages, gender, race, and IUGR status did not differ significantly between the two patient groups (Table 2). There were similar rates of treatment with lipid lowering at drug start and similar lengths of drug treatment. Doses of phenobarbital and ursodiol were within standard of care ranges. All doses of phenobarbital were given intravenously and all doses of ursodiol were given enterally. The direct bilirubin at start of drug therapy was similar between the two groups. For the first course only analysis, 29% of infants were on 70/kg feeds and 16% of infants were on 140/kg feeds in the phenobarbital group. 89% of infants were on 70/kg feeds and 62% of infants were on 140/kg feeds in the ursodiol group. For the all courses analysis, 43% of infants were on 70/kg feeds and 30% of infants were on 140/kg feeds in the phenobarbital group. 86% of infants were on 70/kg feeds and 60% of infants were on 140/kg feeds in the ursodiol group.

**Table 2:** Patient variables by drug (first course of drug therapy)

	Phenobarbital (*N* = 37)	Ursodiol (*N* = 31)	*p*-value
Gestational Age at Birth (%)			0.7628
22–28 weeks	38	45	
28–34 weeks	40	32	
> 34 weeks	22	23	
Male (%)	57	71	0.2261
Race (%)			0.1439
Caucasian	58	61	
African American	20	32	
Other	22	7	
IUGR (%)	19	32	0.2058
On Restricted Lipids at start of drug therapy (%)	46	39	0.5479
Length of Drug Course ^a^	17 (13,38)	17 (12,32)	0.4557
Dose (mg/kg/day) ^a^	4.48 (3.84, 5.05)	27.43 (22.39,29.00)	n/a
Direct Bilirubin at start of drug therapy			0.8458
mg/dL^b^	7.1 (3.98)	6.9 (4.92)	
umol/L ^b^	121.1 (68.1)	118.0 (84.2)	

^a^: Median (lower quartile, upper quartile); ^b^: Mean (SD). IUGR = in utero growth restriction

**Fig. 1:**
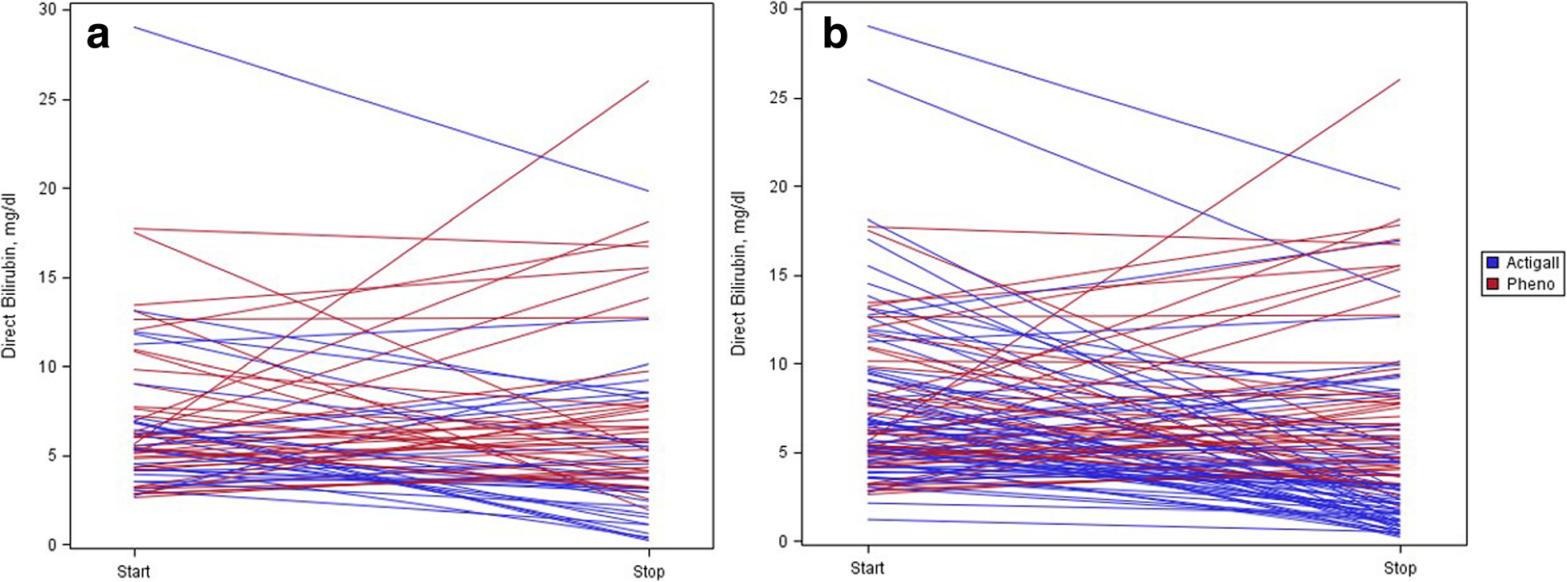
Change in Direct Bilirubin by Drug Treatment. **a** First Course Only: Direct Bilirubin improved in 37% of phenobarbital courses and 70% of ursodiol courses. **b** All Drug Courses: Direct Bilirubin improved in 39% of phenobarbital courses and 77% of ursodiol courses

The results of the drug comparisons are displayed in Table 3. In the primary analysis using the first course of drug therapy only, ursodiol was significantly more effective in reducing direct bilirubin than was phenobarbital (− 1.89 vs + 0.76 mg/dl; − 33.33 vs + 13.0 umol/L, *p*-value .03), even after controlling for baseline cholestasis severity, IUGR status, and lipid lowering therapy (− 2.16 vs + 0.27 mg/dl; − 36.94 vs + 4.62 umol/L, *p*-value .03). In the analysis including all treatment courses, ursodiol was again significantly more effective in reducing direct bilirubin than was phenobarbital (− 3.96 vs + 0.28 mg/dl; − 67.73 vs + 4.79 umol/L, *p*-value <.01). Figure 1 is a spaghetti plot of change in direct bilirubin for (a) first courses, and (b) all drug courses.

**Table 3:** Cholestasis outcomes by drug treatment

	Phenobarbital	Ursodiol	*p*-value
Univariate analysis (first course only)			
Change in direct Bilirubin			0.03
mg/dl	+ 0.76	−1.89	
umol/L	+ 13.0	−32.3	
Direct Bili at end of drug therapy			0.02
mg/dl	7.84	4.98	
umol/L	134.1	85.2	
Adjusted for direct Bilirubin at start of drug therapy			
Change in direct Bilirubin			
First course			0.01
mg/dl	+ 0.81	−1.95	
umol/L	+ 13.85	- 33.35	
All Courses			<0.01
mg/dl	+ 0.66	−3.63	
umol/L	+ 11.29	−62.08	
Direct Bili at end of drug therapy (mg/dl)			
First course			0.01
mg/dl	7.79	5.03	
umol/L	133.24	86.03	
All Courses			<0.01
mg/dl	8.27	3.98	
umol/L	141.45	68.07	
Adjusted for direct Bilirubin at start of drug therapy, IUGR and limited lipids			
Change in direct Bilirubin			
First course			0.03
mg/dl	+ 0.27	−2.16	
umol/L	+ 4.62	−36.94	
All Courses^≠^			
			< 0.01
mg/dl	+ 0.28	−3.96	
umol/L	+ 4.79	−67.73	
Direct Bili at end of drug therapy (mg/dl)			
First Course			0.03
mg/dl	7.25	4.83	
umol/L	124.0	82.61	
All Courses^a^			< 0.01
mg/dl	7.89	3.65	
umol/L	134.95	62.43	

The first course analysis compares 37 phenobarbital to 31 ursodiol courses. The all course analysis compares 46 phenobarbital to 66 ursodiol courses. ^a^The all course analysis was also adjusted for a binary variable accounting for previous phenobarbital or ursodiol exposure in prior 14 days

## Discussion

This is the first study comparing effectiveness of ursodiol vs phenobarbital for the treatment of cholestasis in infants. In a real world NICU population, our results show that phenobarbital, on average, causes no improvement in direct bilirubin. Ursodiol is associated with more improvement in direct bilirubin and a lower direct bilirubin at the end of drug therapy. Although it is not typical to compare an IV formulation (phenobarbital) to an enteral formulation of another drug (ursodiol), we feel this comparison is meaningful because these are the two drugs used to treat cholestasis in the NICU in clinical care and the formulations in which they are given for the same diagnosis. A randomized controlled trial of these off-label, off patent medications is unlikely to occur, and thus a comparative cohort study is the current best evidence we can provide.

Ursodiol is the standard of care drug for cholestasis management, and comparing this to phenobarbital, a drug used without evidence of efficacy, is the only way to ascertain phenobarbital’s utility for this indication. Many clinicians use phenobarbital for cholestasis when an infant is critically ill and not yet enterally fed or on low percent of enteral feeds, while awaiting the ability to use ursodiol once the infant has improved enteral feeding tolerance. T our knowledge, this is the first data which shows a lack of efficacy of phenobarbital in the treatment of neonatal cholestasis, and suggests that clinicians may consider avoiding phenobarbital treatment and waiting for sufficient enteral feeds to start ursodiol as the treatment of choice.

Despite lack of evidence, clinicians still use phenobarbital to treat neonatal and infant cholestasis. In a retrospective cohort study of neonates receiving phenobarbital for neurologic treatment (5 mg/kg/day), 66% of phenobarbital-treated infants developed cholestasis, as opposed to 33% of untreated controls [13], providing indirect evidence for lack of utility in cholestasis. In studies of drug-augmented hepatobiliary scintigraphy, results are mixed in regards to phenobarbital efficacy. When 50 infants with non-excreting phenotype on HIDA scan were randomized to phenorarbital, ursodiol or placebo, there was no increase in biliary excretion with any treatment [14]. In a direct comparison of the two drugs, the specificities of diagnosis of biliary atresia on drug-augmented scintigraphy were 80% for phenobarbital and 96.6% for ursodiol [15].

In addition to lack of efficacy, there is animal data which supports the role of phenobarbital as a neurotoxin. Normal rat pups equivalent in developmental age to human newborns were exposed to phenobarbital and areas of brain were examined for injury at 24 h. As compared to control, phenobarbital-treated rats had significantly increased levels of neuronal apoptosis in multiple brain regions [10]. In a different study, normal rat pups exposed to a single dose of phenobarbital at human newborn equivalent age exhibited impaired physiologic maturation in synaptogenesis among surviving neurons, suggesting that early life exposure can potentially impact cognitive and behavioral outcomes [16]. Rats with a single exposure to phenobarbital during a critical window of brain development showed schizophrenia-like behavioral abnormalities as adults, suggesting that early neuronal injury and disordered synaptogenesis can predispose to psychiatric illness [17]. When used for cholestasis, phenobarbital is dosed daily for multiple days (median of 17 days in our cohort), and this prolonged dosing during a critical period of brain development may place infants at risk for neuronal abnormalities.

As with all retrospective cohort studies, our study has some limitations. First, since other markers of hepatic injury (AST/ALT, GGT, and hepatic ultrasound) are not closely followed clinically in many infants with cholestasis, direct bilirubin was the only universally available marker of cholestasis available as a surrogate marker of disease. Second, most infants with cholestasis in our cohort were treated with multiple courses of medications for cholestasis. To account for the effects of a prior drug course influencing the clinical results of a subsequent drug course, we did a primary analysis comparing only first courses of drug therapy. Third, given the critically ill condition of the patient population, most infants are not enterally fed initially and are treated with intravenous phenobarbital until on sufficient enteral feeds to tolerate ursodiol (an enterally dosed medication). This is the reality of the care of these infants and is reflected in the study results. We understand that enteral feedings could have confounded the association between ursodiol therapy and improvement in direct bilirubin because enteral feeds are themselves associated with improvement in cholestasis. Although enteral feeds likely augment the improvement of direct bilirubin seen with ursodiol, it seems unlikely that this accounts for the entire difference we observed between the two medications.

Strengths of this research include the diversity of medical diagnoses among the patients included in the cohort, making the results widely generalizable for NICU patients. Additionally, the narrow range of phenobarbital and ursodiol daily doses minimized the effect of drug dosing variability on the outcome. Lastly, we were able to account for the other main treatment modality for cholestasis, IV lipid limiting, in the comparative analysis.

There are potential new drugs for the treatment of cholestasis, such as obethicolic acid, a farnesoid C receptor (FXR) agonist, which regulates a wide variety of genes critical to bile acid synthesis and transport. FXR agonism decreases synthesis of bile acids in hepatocytes and increases transport of bile acids out of the cells, overall reducing the cytotoxic injury to hepatocytes. This drug was FDA approved this drug in May 2016 for the treatment of primary biliary cholangitis in combination with ursodiol. The pharmacokinetics, safety and efficacy of this drug have not been studies in neonates.

## Conclusion

In conclusion, our study is the first to show that phenobarbital, when dosed over many days, does not significantly improve cholestasis in infants as compared to ursodiol. The burgeoning evidence for neurotoxicity in the developing brain associated with phenobarbital use may steer a treating clinician towards the use of ursodiol as the drug of choice for treatment of cholestasis in neonates.

## Abbreviations

ALT: Alanine aminotransferase
ANOVA: Analysis of variance
AST: Aspartate aminotransferase
CAR: Constutuitive androstane receptor
CDH: Congenital diaphragmatic hernia
DBili: Direct bilirubin
dL: deciliter
ECMO: Extracorporeal membrane oxygenation
GGT: Gamma glutamyl transferase
IUGR: In utero growth restriction
IV: Intravenous
NEC: Necrotizing enterocolitis
NICU: Neonatal intensive care unit
